# Reply to `Comment on `Clinical significance of BRAF non-V600E mutations on the therapeutic effects of anti-EGFR monoclonal antibody treatment in patients with pretreated metastatic colorectal cancer: the Biomarker Research for anti-EGFR monoclonal Antibodies by Comprehensive Cancer genomics (BREAC) study''

**DOI:** 10.1038/s41416-018-0040-y

**Published:** 2018-03-22

**Authors:** Eiji Shinozaki, Takayuki Yoshino, Katsuya Tsuchihara

**Affiliations:** 1 Department of Gastrointestinal Oncology, Cancer Institute Hospital of Japanese Foundation for Cancer Research, Tokyo, Japan; 2 Department of Gastroenterology and Gastrointestinal Oncology, National Cancer Center Hospital East, Kashiwa, Japan; 3 Division of Translational Genomics, Exploratory Oncology Research and Clinical Trial Center, National Cancer Center, Chiba, Japan

**Keywords:** Colorectal cancer, Biomarkers

In a recent comment on our manuscript “Clinical significance of BRAF non-V600E mutations on the therapeutic effects of anti-EGFR monoclonal antibody treatment in patients with pretreated metastatic colorectal cancer: the Biomarker Research for anti-EGFR monoclonal Antibodies by Comprehensive Cancer genomics (BREAC) study”,^1^ Dankner and Rose (2018)^2^ suggested insightful and important limitations of our study. Dankner and Rose discussed that it remains to be seen whether all *BRAF* non-V600 mutations in mCRC tumours are equally predictive of non-response to EGFR inhibitors, based on the recent classification of *BRAF* mutation^3^ as well as their original data. They reported that some class 3 *BRAF* mutants (i.e., G446V) were sensitive to anti-EGFR antibodies in both clinical and preclinical models. We agree with the speculation that it is not the same of *BRAF* non-V600 mutations, and we concluded that ‘certain’ *BRAF* non-V600E mutations might contribute to a lesser benefit of anti-EGFR monoclonal antibody treatment. We also consider the hypothesis of the difference in RAS dependencies of class 3 *BRAF* mutations as intriguing.

We would like to address the points raised by Dankner and Rose in their comment regarding our data^2^. They pointed out the differences in overall survival (OS) between the two studies^4^ and our cohort. Firstly, we would like to highlight that the definition of OS was different; survival was calculated from the time of first diagnosis of metastatic disease in Jones et al. and from the start of later line treatment in our study. Furthermore, our cohort only consisted of patients that survived until later line treatment. Truly aggressive disease cases might not complete later line treatment. One of the explanations for their question might be the inclusion of very selective patients in our later line treatment cohort; patients with highly aggressive disease and those harbouring *BRAF* V600E were inevitably excluded.

We re-summarised the individual PFS data of *BRAF* non-V600E in Table 1. One case with D495G, class 3, showed long stable disease (SD) as efficacy. However, 4 of 7 were not reported in *BRAF* categories by Yao et al., regardless of their statement that the majority of *BRAF* non-V600 mutations in CRC are class 3 mutations. Furthermore, we do not have enough data in Asian populations and the racial differences in *BRAF/KRAS* mutation rates are well known;^5^ this was discussed in our discussion. In our cohort, only 2 cases were categorised as class 3, and the kinase activity of the other unclassified cases was classified high or intermediate. Regarding the response rate, other than the sample size, the proportion of *BRAF* class category might be affected for efficacy in this cohort.

**Table 1:** Individual data of patients harbouring the BRAF non-V600E mutation

ID	Amino acid variation	Kinase activity	PFS (mo)	Class^a^
GQ0XS	G469A	High	2.8	NR
GLCH7	L485F	Intermediate	2.1	NR
SC12PCQ3IA02	Q524L	Intermediate^b^	2.3	NR
G9OJR	L525R	High^b^	4.0	NR
GQ4U5	D594G	Impaired	6.6	III
GUZG7	D594G	Impaired	2.4	III
GS3A5	V600R	High	2.1	I

*mo* month, *NR* not reported, *PFS* progression-free survival

^a^Class (by Yao et al, 2017)

^b^Our reported data

We recognise the limitations of this study; a retrospective study with a small number of subgroups of *BRAF* non-V600E mutations. Further investigation in much larger scale data set from clinical trials such as randomised control trials is necessary to conclude the significance of anti-EGFR antibody treatment for each subtype of *BRAF* non-V600E mutational variants.
